# Job Burnout and Related Factors among Health Sector Employees 

**Published:** 2019-10

**Authors:** Elham Bazmi, Abbas Alipour, Mohamad Taghi Yasamy, Ali Kheradmand, Soosan Salehpour, Soheila Khodakarim, Hamid Soori

**Affiliations:** 1 Safety Promotion and Injury Prevention Research Center. (SBMU Employees’ Health Cohort center), Shahid Beheshti University of Medical Sciences, Tehran, Iran.; 2 Department of Psychiatry, Taleghani Hospital, Shahid Beheshti University of Medical Sciences, Tehran, Iran.; 3 Occupational Medicine Specialist, Poursina Occupational Medicine Services Center, Tehran, Iran.; 4 Department of Epidemiology, School of Public Health and Safety, Shahid Beheshti University of Medical Sciences, Tehran, Iran.

**Keywords:** *Associated Factors*, *Burnout*, *Employees*, *Health Care*

## Abstract

**Objective:** Job burnout can cause physical and psychological damage and reduce job efficiency, especially in difficult jobs such as health care fields. This study aimed to assess the association between the level of job burnout and some contributing factors among health care providers in Iran.

**Method** **:** This study was performed on the data derived from 1807 participants from the first phase of the employees’ health cohort in 2017-2018. The data were collected using as a self-administrated tool utilizing Maslach Burnout Inventory. The questionnaire scores ranged from never to everyday, with 3 levels of burnout as well as burnout itself; then, the scores were categorized as low, moderate, or high. Ordinal logistic regression model was used to adjust ordinal dependent variables.

**Results: **The mean score of the total burnout was 16.5±7.77 and was associated with work experience and age group (p < 0.001). The components of burnout consisted of emotional exhaustion (8.9± 9.0), depersonalization (23± 2.9), and personal accomplishment (34± 8.6). Emotional exhaustion was related to sex (less among males, OR=0.48) and type of job (less among officials compared to health care staff, OR=0.488). Composite burnout was more common among younger staff (OR= 3.85). Depersonalization was associated with duty shift workers (OR=2.42).

**Conclusion: **Job burnout is a major concern, and lack of personal accomplishment, as a component of burnout, was highly prevalent among Iranian health care employees. Being a single woman, health care provider, and having more than 20 years of work experience with a duty shift were contributing factors for burnout experience. Monitoring symptoms of burnout and its associated factors in the workplace and proposing an alternative organizational and behavioral system and sharing it with relevant authorities may help prevent or reduce job burnout and its deleterious effects.

Job burnout was introduced in 1970 as a negative work-related state of mind which occurred due to the presence of job demands such as work overload, prolonged working hours, lack of fairness, job conflict, lack of social support from colleagues or supervisor, and lack of decision-making authority (1, 2). Some of the consequences of job burnout were absenteeism, low morale or personnel deterioration, stress, anxiety, pychosomatic complains, sleep disturbance, and poor organizational commitment (2).

One of the main factors in the generation of burnout was the type of work that an individual had to perform. Burnout had been mostly used to study workers at human services organizations, such as school teachers, social workers, and health care staff (3).

Nature of patient care, and high emotional exhaustion (4 , 5 , 6). Burnout among health care providers causes increasing absence and reduces efficiency at work. Therefore, identification and prevention of burnout plays an important role in improving the quality of provided services (7).

The employees in the health sector experience higher rates of work burnout than other jobs due to their greater exposure to stressful organizational demands, grueling nature of patient care, and high emotional exhaustion (4‏, 5 and 6. Burnout among health care providers causes increasing absence and reduces efficiency at work. Therefore, identification and prevention of burnout plays an important role in improving the quality of provided services (7).

Burnout contains 3 dimensions: (1) emotional exhaustion (core dimension of burnout), which consists of chronic exhaustion and sleep disorder; (2) depersonalization, which includes negative reaction without any feeling and with an extreme indifference to the recipients of services; (3) sense of lack of personal accomplishment, which reduces the sense of merit and success (8, 9, 10).

Burnout among employees has been studied mostly in Europe, the United States, and the Middle East (10, 11). Various demographic factors such as age, gender, type of occupation, management style, lack of social support, lack of success, lack of career promotion opportunities, experience, working overtime, undesirable working conditions, and shiftwork could lead to the feelings of burnout. The results of studies usually showed a negative association between burnout and job satisfaction, which was influenced by organizational structure; also, a positive relationship was found between work experience and lack of social support (12-15). 

However, only a limited number of studies have been conducted to study various dimensions of job burnout and its associated factors in different employees of universities of medical sciences. Therefore, the present study aimed to assess the level of burnout among the Medical staff working in Shahid Beheshti University of Medical Sciences, Tehran, Iran, and to evaluate the main demographic factors associated with burnout among them.

Iran has been going through different phases of economic and social upheavals and the impact of sanctions has been adding up to the severity of job burnout. This study aimed to assess the association between the level of job burnout and some contributing factors among health care providers in Iran. 

## Materials and Methods

This study was conducted from February 2017 to December 2018. A prepilot and pilot study were preformed to test the instruments and feasibility of the study. 

The methods and proposal of this study were reviewed, informed consent letter was received from participants, and all were approved by the ethical and research committee of Shahid Beheshti University of Medical Sciences (30 October 2016 No: 178).

The instruments used in the study consisted of a 30-item demographic questionnaire and the Maslach Burnout Inventory (MBI) that were made available in participants’ workplaces (1, 3). The reliability and validity of the questionnaire had already been confirmed in Iran (9, 17, 20). The reliability of this questionnaire was acceptable (alpha=0.85) and alpha Cronbach dimensions of burnout was 0.91, 0.92, and 0.77 respectively. To determine the validity of the questionnaire, it was translated to Persian and an expert panel confirmed its face and content validity. Validity of the questionnaire was 0.90 for demographic characteristics. The demographic questionnaire included questions on age, gender, marital status, number of children, the place that the participant worked, number of years working, educational level, and other demographic variables. 

MBI version was the 22-item inventory, with 3 subscales (9 questions on emotional exhaustion, 5 on depersonalization, and 8 on the sense of lack of personal accomplishment). Items of burnout were scored using Likert scale, ranged from 0 to 6 (never, sometimes in a year, once a year, sometimes in a month, sometimes in a week, once a week, and every day). The emotional exhaustion (EE) subscale measured feeling of being emotionally overextended, long-term exhaustion at work, and sleep disorder. The depersonalization (DP) subscale measured feeling callous toward patients, students, other employees, and impersonal response toward recipients of the services, treatment, and instruction. The reduced personal accomplishment (PA) measured the extent to which an employee experienced feelings of incompetence in the job. In a similar study in Iran, the reliability of this questionnaire was found to be acceptable (alpha=0.85), and alpha Cronbach dimensions of burnout was 0.91, 0.92, and 0.77 respectively (17). 

Emotional exhaustion (EE) scores below 19 indicated low degree, 19-26 moderate, and above 26 as high EE. DP scores below 6 were considered low, 6-9 moderate, and higher than 9 high. Personal accomplishment scores of 34-39 indicated moderate and lower accomplishment and scores higher than this range were considered as low and high (1, ‏^3^). After explaining the goals of the study, MBI and the demographic questionnaires were completed by the participants as self-report. More details about the methods of the original study can be find in reference 16.

***Participants***

The study population consisted of 1807 employees whose data were derived from the first phase of the employees’ health cohort in Shahid Beheshti University of Medical Sciences, Tehran, Iran, and who were willing to participate in this investigation and able to complete the questionnaire (16). The employees’ health cohort was selected using stratified random sampling and included physicians, dentists, pharmacists, nurses, administrative employers, and unskilled employees. The participants aged 18 to 65 years and had at least one year of job experience and employees with more than one-month job absence at the time of data collection were excluded. All participants were invited to the study place and 2 trained clinical psychologists (Master’s degree) collected data.

***Data Analysis***

The collected data were analyzed using inferential statistics such as Spearman's correlation coefficient, independent t test, and the chi-squared test. Since burnout is an ordinal variable, ordinal logistic regression was used. 

All predictors that had been associated with burnout scale on the simple or single model with a p-value of less than to 0.25 were included in the ordinal logistic regression model of multiple analysis. Likelihood ratio test was used to compare the final model with other models. The crude and adjusted odds ratios and 95% confidence intervals were computed as well. P values less than 0.05 were considered statistically significant. Statistical analysis was performed using SPSS version 24.

## Results

Among 2101 employers (response rate 86%), 1807 filled the inventory completely. The average age of participants was 41.49± 8.54 years and 75.9% of primary health care providers participating in the study were married. Their average work experience was 15.8± 4.0 years. The median of the number of children was 1. Most participants (72%) did not have duty shift. The frequency distribution of demographic factors among employees in Shahid Beheshti University of Medical Sciences of Iran is shown in Table 1.

Frequency of burnout domains and total burnout among employees through MBI is shown in Table 2. 

The mean score of burnout was 7.7‏ ±16.5 and the mean score of burnout components was 8.9±9.0, 23±2.9, and 34±8.6, respectively, for emotional exhaustion, depersonalization, and reduced personal accomplishments. 

Table 3 demonstrates some relationship between components of burnout and demographic variables. Emotional exhaustion was associated with marital status, education level, experience, and number of children. Depersonalization was related to sex and years of work experience. Finally, there was a significant inverse association between reduced personal accomplishment and sex, marital status, second jobs, and number of children. 

The results of the analysis of the variables showed a significant relationship between total burnout, marital status experience, and the number of children (P < 0.05), whereas the relationship between burnout and other variables, including age, sex, level of education, second job, type of employment, and duty shift were not significant. 

The result of the ordinal regression model showed that emotional exhaustion was related to sex (less among males OR=0.48) and type of job (less among officials compared to health care staff OR=0.488). Composite burnout was more common among younger staff (OR=3.85). Depersonalization was associated with duty shift workers (OR=2.42). Sex, shift duty, and education level remained in the final model and examined using Maximum Likelihood ratio (LR test) in 3 subscales of burnout (Table 4).

**Table 1:** Frequency Distribution Demographic Factors among Employees in Shahid Beheshti University of Medical Sciences of Iran

**Variables**	**Number of participants**	**Percent**
Age:		
Less than 30	34	10.9
30-50	229	73.4
Over 50	49	15.7
Sex:		
Male	151	48.4
Female	161	51.6
Marital status:		
Married	247	79.2
Single	50	16.0
Widow	6	1.9
Divorced	9	2.9
Educational level:		
< Diploma	57	18.3
Under graduate	174	55.8
>Bachelor	81	26.0
Second job:		
Yes	81	26.0
No	227	72.8
Missing	4	1.3
Job experience:		
<10 year	121	38.8
10-20 year	114	36.5
>20 year	77	24.7
Type of job:		
Official	120	38.5
Laborer	83	26.6
Health care	105	33.7
Missing	4	1.3
Number of children:		
<1	89	28.5
1-3	210	67.3
>3	13	4.2
Duty shift:		
Yes	91	29.2
No	217	69.6
Missing	4	1.3

**Table 2:** Frequency of Burnout Domains and Total Burnout among Employees in Shahid Beheshti University of Medical Sciences, Iran

**Burnout domains**	**% Low**	**%Moderate**	**% High**
Emotional exhaustion	83	13.1	3.2
Depersonalization	83	12	4.8
Reduced personal accomplishment	37	36	26
Total burnout	3.2	85.9	70.3

## Discussion

This study showed that most of health care employees have experienced low level of emotional exhaustion, depersonalization, and reduced personal accomplishments, which were different from some other studies and similar to other researches (3, 18-20). There was a significant correlation between burnout dimensions and some related factors in employers of Shahid Beheshti University of Medical Science, Iran.

According to the results, most of employees (85.9%) had experienced a moderate level of burnout. However, in the study of Abdi et al that used MBI, 2 dimensions of burnout (emotional exhaustion and depersonalization) were moderate, but reduced personal incompetence was at high level. Hirut et al reported that 54.8% of nurses had faced job burnout, which was different from our results due to the urban nature of their study, but the present study was comparable with the study conducted by Matin BK et al which revealed that 40.2% of health staff had experienced burnout (21, 22).

The 3 levels of emotional exhaustion in the 30-50 age group was more frequent than in other groups, which is similar to other studies. Therefore, by increase in age, the work experience increased and employees suffered less burnout compared to the younger. However, the results of this study showed no significant relationship between age and emotional exhaustion possibly because as the employees have more years of experience they feel they have a safer job position, which is similar to Amiri et al and Garrosa et al findings (17, 23‏).

The results indicated no significant association between sex and EE, which is consistent with the results of other research reports but inconsistent with the results of Williams et al (14, 24). Many experts attributed the high emotional exhaustion to additional responsibility of females at home and accepting the similar role as wives, mothers, and going through hormonal changes as well (25).

There was a significant association between emotional exhaustion, depersonalization, and marital status that was similar to Rashidi et al (2012) and Iglesias et al (2010). They mentioned that married individuals had more personal incompetence and lack of control over additional responsibility. The reason was support from family and increasing motivation for their future (5, 26).

The results of the present study showed a significant relationship between job experience and EE that was inconsistent with Iglesias and Boyas et al (5, 27). The employees who had fewer than 10 years of experience in the University of Medical Science were 2.07 times less likely to have emotional exhaustion than those who had 10-20 working years, and they experienced 1.72 times less emotional exhaustion than those who had greater than 20 years. These results were the same as reports of Hirut et al, which may be due to the presence of workload and job conditions during the time of the study (21, 28, 29).

Those with lower than diploma educational levels were 1.57 times more vulnerable to emotional exhaustion as compared to those holding graduate degrees. These findings were similar to the reports of previous studies done by other researches (30, 31). The results could be attributed to expectations of a generally well-educated person. Schandenhofer et al found that having a low level of education was regarded as an important factor in burnout of females which was congruent with what was discovered in this study (29).

The results indicated that health care workers suffered from the highest levels of emotional exhaustion, depersonalization, and reduced personal accomplishment compared to administrative employees and unskilled workers. These results were consistent with those of other studies (31, 32). The high level of EE among health care employees may be associated with the high workload in public health organizations. It appears that many factors, including more accountability, the pressure of work, shortage of equipment and facilities, had important roles in EE.

This study showed that emotional exhaustion was more among health care staff because of their sex characteristics and exposure to more emotional situations. However, this result was not consistent with that of Demir et al. They found that females and nurses had a significant association with increased emotional exhaustion (33).

The findings of this study indicated a significant relationship between depersonalization with sex and duty shift work; however, there was no significant relationship between DP and marital status, age, and education level, which is the same as the results of William et al, and Iglesian and Boyas et al (5, 24, 27).

Depersonalization may reduce level of psychological connection with others in the workplace and has serious implications for the quality of health care services and level of commitment to the organization.

In this research, a significant relationship was found between lack of personal accomplishment, marital status, having a second job, and number of children. Piko also found a correlation between sex and reduced personal accomplishment, but no association with age, which is consistent with some part of the recent findings (14). However, Garrosa et al and Iglesia et al did not observe a significant relationship between sex and reduced personal accomplishment (5, 23).

In the present study, participants with medium length of stay on the job (10-20 years) and more than 20 years had a significantly higher level of reduced personal accomplishment, which could be due to uncertainties about the meaningfulness of their work and lack of satisfaction with their job. These findings were similar to those of other studies (34).

The results showed that among health care staff, the high feelings of reduced personal accomplishment may be a consequence of poor infrastructure and inadequate health care supplies.

Also, a significant relationship was found between duty shifts with depersonalization and reduced personal accomplishment, which supports Rezaei et al study (35). They suggested that working in night shifts would change the time of sleep and could contribute to job burnout. 

Also, this study showed a significant statistical correlation between job satisfaction and frequency and intensity of EE (p=0.014). On the other hand, job satisfaction reduction can cause constant stress, feeling of psychological exhaustion, and increased emotional exhaustion, which is similar to results of Rezaei et al (35).

This study also srevealed a significant relationship between total burnout with experience and age, but no significant association between sex, type of job, duty shift, education level, and marital status, which is consistent with the findings of Iglesias et al, Boyas et al, and Schadenhofer et al (4, 26, 29). However, the present findings were not in line with the results of Rashidi et al and Talei et al who reported a relationship between marital status and burnout (9, 26). It seems that work experience of employees was an important factor which helps them to tolerate problems that lead to burnout.

The present research was conducted in Shahid Beheshti University of Medical Sciences of Iran that covers a diverse range of occupational groups and different demographic characteristics. This was in fact the strength of this study (16). However, this study had some limitations. MBI is a gold standard to measure job burnout, but using self-reporting questionnaires, including MBI, may potentially cause response bias through social desirability. Although the response rate was acceptable, the cross sectional nature of the study could not allow making causal claims. However, data coming from future measurements in the course of the cohort study can be more fruitful in this respect. 

**Table 3:** Comparing Frequency (%) Distribution of Burnout Dimensions Based on Demographic Variables among Employers of Shahid Beheshti University of Medical Science, Iran

**Variable**	**Burnout Dimensions**									
	**Emotional exhaustion**				**Depersonalization**			**Reduced personal ** **accomplishment**		
	**Low**	**Moderate**		**High**	**Low**	**Moderate**	**High**	**Low**	**Moderate**	**High**
Age:										
Less than 30	27(79.4)	6(17.6)		1(2.9)	26 (76.5)	4 (11.8)	4 (11.8)	15 (44.1)	12(35.3)	7(20.6)
30-50	188(82.8)	31(13.7)		8(3.5)	190 (83.7)	29(12.8)	8 (3.5)	85 (37.4)	82(36.1)	60(26.4)
Over 50	44(89.8)	4(8.2)		1(2.0)	42 (85.7)	4(8.2)	3 (6.1)	16 (32.7)	17(34.7)	16(32.7)
P value	0.07				0.06			0.075		
Sex:										
Male	134(88.7)	13(8.6)		4(2.6)	127(84.1)	16(10.6)	8 (5.3)	57(37.7)	57(37.7)	37(24.5)
Female	125(78.6)	28(17.6)		6(3.8)	131(82.4)	21(13.2)	7 (4.4)	59(37.1)	54(34.0)	46(28.9)
P. value	0.134				0.02			0.032		
Marital status:										
Married	208(84.6)	30(12.2)		8(3.3)	210(85.4)	26(10.6)	10(4.1)	92(37.4)	88(35.8)	66(26.8)
Single	38(77.6)	9(18.4)		2(4.1)	37 (75.5)	7(14.3)	5(10.2)	19(38.8)	17 (34.7)	13(26.5)
Widower	5(83.3)	1(16.7)		0(0.0)	5(83.3)	1(16.7)	0 (0.0)	2 (33.3)	2 (33.3)	2(33.3)
Divorced	8(88.9)	1(11.1)		0(0.0)	6(66.7)	3(33.3)	0 (.0)	3 (3.3)	4 (44.4)	2(22.2)
P. value	0.046				0.113			0.001		
Education level:										
< Diploma	84(84.0)	12(12.0)		4,4.0)	80,80.0)	15,15.0)	5,5.0)	49,49.0)	31,31.0)	20,20.0)
Under graduate	103(79.8)	20(15.5)		6 (4.7)	104(80.6)	16(12.4)	9 (7.0)	55(42.6)	43 (33.3)	31(24.0)
>Bachelor	72 (88.9)	9 (11.1)		0(0.0)	74(91.4)	6(7.4)	1 (1.2)	12(14.8)	37(45.7)	32(39.5)
P. value	0.047				0.109			0.2		
Second job:										
Yes	72 (88.9)	8 (9.9)		1(1.2)	67(82.7)	11(13.6)	3 (3.7)	27(33.3)	33 (40.7)	21(25.9)
No	183 (81.3)	33 (14.7)		9(4.0)	188(83.6)	26(11.6)	11(4.9)	89(39.6)	74 (32.9)	62(27.6)
P. value	0.101				0.15			0.033		
Job experience:										
<10 year	101 (84.2)	16 (13.3)		3(2.5)	99(82.5)	14(11.7)	7 (5.8)	49(40.8)	47(39.2)	24(20.0)
10-20 year	92 (80.7)	18 (15.8)		4(3.5)	93(81.6)	17(14.9)	4 (3.5)	42(36.8)	36(31.6)	36(31.6)
>20 year	66 (86.8)	7 (9.2)		3(3.9)	66(86.8)	6(7.9)	4 (5.3)	25(32.9)	28(36.8)	23(30.3)
P. value	0.013				0.037			0.095		
Type of job:										
Official	104 (87.4)	13 (10.9)		2(1.7)	108(90.8)	6(5.0)	5 (4.2)	33(27.7)	46(38.7)	40(33.6)
Laborer	71 (85.5)	8 (9.6)		4(4.8)	67(80.7)	12(14.5)	4 (4.8)	43(51.8)	26(31.3)	14(16.9)
Health care	80 (76.9)	20 (19.2)		4(3.8)	80(76.9)	19(18.3)	5 (4.8)	40(38.5)	35(33.7)	29(27.9)
P. value	0.11				0.15			0.099		
Number of children:										
<1	71 (80.7)	14 (15.9)		3(3.4)	69(78.4)	11(12.5)	8 (9.1)	35(39.8)	28 (31.8)	25(28.4)
1-3	177 (84.7)	25 (12.0)		7(3.3)	178(85.2)	25(12.0)	6 (2.9)	74(35.4)	80(38.3)	55(26.3)
>3	11 (84.6)	2 (15.4)		0(0.0)	11(84.6)	1(7.7)	1 (7.7)	7 (53.8)	3 (23.1)	3(23.1)
P. value	0.046				0.087			0.009		
Duty shift:										
Yes	72 (75.5)		19(19.8)	5(5.2)	71(74.0)	19(19.8)	6(6.3)	41(42.7)	32(33.3)	23(24.0)
No	183 (87.1)		22(10.5)	5(2.4)	184(87.6)	18(8.6)	8(3.8)	75(35.7)	75(35.7)	60(28.6)
P. value	0.135				0.168			0.061		

**Table 4:** The Association between Burnout and Associated Factors among Employees of Shahid Beheshti University of Medical Science, Iran by Multivariate Analysis Using Ordinal Logistic Regression Model

		**Composite ** **Burnout**	**Emotional ** **exhaustion**	**Depersonalization**	**personal ** **accomplishment**
**Variable**		**OR(95%CI)**	**OR(95%CI)**	**OR(95%CI)**	**OR(95%CI)**
Sex:	Male		0.48(0.25-0.88)		
	Female		1		
Job Experience:	<10 Years	2.78(1.44-5.37)	1.584(2.273-0.89)	1.66(0.39-1.12)	
	10-20 Years	1.65(8.88-3.12)	1.483(2.238-0. 73)	0.93(0.84-1.5)	
	>20 Years	1	1	1	
Type of job:	Official		0.488(0.23-0.97)	0.35(0.164-0.754)	1.46(0.90-2.38)
	Laborer		0.58(0.27-1.25)	0.80(0.399-1.64)	1.55(0.96-1.8)
	Health care		1	1	1
Duty shift:	Yes			2.42(1.31-4.48)	0.75(0.48-1.8)
	No				
Education level:	< Diploma		1.57(0.658-3.77)	2.62(1.06-6.6)	0.32(0.2-0.52)
	Under graduate		2.081(0.922-4.69)	2.61(1.07-6.35)	0.51(0.32-0.66)
	>Bachelor		1	1	1
Marital status:					
	Married Single			0.398(0.1-1.5) 0.781(0.177-3.44)	
	Widower			0.439(0.036-5.43)	
	Divorced				
Age:	Less than 30	3.85(1.31-11.39)		0.57(0.25-1.30)	
	30-50	2.44(1.26-4.71)		0.77(0.43-7.37)	
	Over 50	1			

## Conclusion

This study revealed that most of the participants were experiencing job burnout. Reduced personal accomplishment was highest among employees of the University of Medical Sciences as compared to those in the health services. Being a single woman, health care provider, a postgraduate, and having more than 20 working experience years with a duty shift at the age of 30-50 were contributing factors for burnout. Females health care staff had a higher level of emotional exhaustion, and composite burnout was more common among the younger staff. Therefore, identifying the risks of job burnout and adopting appropriate organizational and behavioral systems by managing the necessary facilities to prevent or reduce job burnout is highly needed. However, the results implied that increasing education level without duty shift will lead to less job burnout, and the future phases of the employees’ health cohort will show these results more clearly. Such data may be used to contribute to better working environments and less burnout, leading to higher levels of well-being and more efficiency at work.
